# Anti-N-Methyl-D-aspartate Receptor Encephalitis: A Severe, Potentially Reversible Autoimmune Encephalitis

**DOI:** 10.1155/2017/6361479

**Published:** 2017-06-18

**Authors:** Cai-yun Liu, Jie Zhu, Xiang-Yu Zheng, Chi Ma, Xu Wang

**Affiliations:** ^1^Department of Neurology and Neuroscience Center, The First Hospital of Jilin University, Changchun 130021, China; ^2^Department of Neurobiology, Care Sciences and Society, Karolinska Institute, 141 86 Stockholm, Sweden; ^3^Department of Neurosurgery, The First Hospital of Jilin University, Jilin University, Changchun 130021, China

## Abstract

Anti-N-methyl-D-aspartate receptor (NMDAR) encephalitis is potentially lethal, but it is also a treatable autoimmune disorder characterized by prominent psychiatric and neurologic symptoms. It is often accompanied with teratoma or other neoplasm, especially in female patients. Anti-NMDAR antibodies in cerebrospinal fluid (CSF) and serum are characteristic features of the disease, thereby suggesting a pathogenic role in the disease. Here, we summarize recent studies that have clearly documented that both clinical manifestations and the antibodies may contribute to early diagnosis and multidisciplinary care. The clinical course of the disorder is reversible and the relapse could occur in some patients. Anti-NMDAR encephalitis coexisting with demyelinating disorders makes the diagnosis more complex; thus, clinicians should be aware of the overlapping diseases.

## 1. Introduction

The encephalitis associated with anti-N-methyl-D-aspartate receptor (NMDAR) antibodies is a recently identified autoimmune disorder with a progressive clinical course and the possibility of effective management and favorable outcome. Since its first description by Dalmau et al. [1], it has gained increasing attention. The California Encephalitis Project enrolling individuals younger than 30 years showed that the frequency of anti-NMDAR encephalitis surpassed that of individual viral etiologies such as herpes simplex type 1 (HSV-1), West Nile virus (WNV), enteroviruses, and varicella-zoster virus (VZV) [2].

The triggers of the disorder comprise viral infections, tumors, and other unknown factors. It is reported that herpes simplex encephalitis (HSE) plays a vital role in triggering the synthesis of anti-NMDAR antibodies [3]. In young adult females, the encephalitis is often accompanied with ovarian teratomas [2, 4], while males and children are also affected, but the presence of a tumor is uncommon [5, 6]. The specific IgG antibodies recognizing the GluN1 subunit of NMDARs result in the receptors' removal from the synapse through a mechanism of crosslinking and internalization, which is titer-dependent and reversible [4, 7, 8]. Clinically, after an influenza-like antecedent infection, the patients manifest with obvious behavioral and psychiatric symptoms, which are commonly accompanied by seizures, memory loss, language dysfunctions, dyskinesias, and impaired consciousness. Additionally, the autonomic instability and hypoventilation are seen in many cases [1, 9]. These symptoms are characteristic; however, misdiagnosis and delayed diagnosis occur commonly. A poor outcome, such as persistent and severe neuropsychiatric deficit, may occur in up to 25% of patients [4, 5]. Relapses are also observed [10, 11]. Despite the complexity and severity of anti-NMDAR encephalitis, full or substantial recovery has been achieved in most patients, who received early diagnosis and prompt multidisciplinary therapy [4]. Here, we aim to review the recent studies on the clinical and laboratory features, diagnosis, and treatments, as well as the mechanisms underlying this disorder.

## 2. Epidemiology

It has been reported that anti-NMDAR encephalitis is the most common antibody-associated encephalitis [12]. Since the original description of anti-NMDAR encephalitis [1], there have been many studies on this disorder. A report from Germany indicated that anti-NMDAR encephalitis represented 1% of young individuals (18–35 years) hospitalized in the intensive care unit (ICU) [13]. In a multicenter study in Korea, of the 721 patients (aged older than 18 years) with encephalitis of unascertained cause, 40 (6%) were diagnosed with anti-NMDAR encephalitis [14]. A prospective study in England recruited 203 patients with symptoms of encephalitis and showed that of 128 cases whose causes were definite, HSV caused the most cases (36, 28%), while only 9 (7%) were attributable to anti-NMDAR encephalitis [12]. Another study reported that anti-NMDAR encephalitis was the leading entity, more than 4 times as frequent as HSV-1, WNV, or VZV [2]. The discrepancy may be due to the different population composition, regions, and heterogenic factors. Nevertheless, there has been no study to report the prevalence rate of the anti-NMDAR encephalitis in a certain region to date. The exact incidence of the disorder is also unknown.

In 2005, anti-NMDAR encephalitis was first identified in four young women who suffered from ovarian teratoma and manifested with acute psychiatric symptoms, decreased level of consciousness, seizures, amnesia, and hypoventilation [15]. In the subsequent years, several reports showed that females were significantly more likely to be involved than males. Between September 2007 and February 2011, of the 32 cases who were identified anti-NMDAR encephalitis in the California Encephalitis Project, 75% (24) were females [2]. In another report including 577 patients, the rate was 81% [11]. In a case-series study containing 51 patients with anti-NMDAR encephalitis from Southwest China, 32 (63%) patients were females [16]. The disorder is more likely to affect younger individuals although patients of all ages can be affected. The median age of 577 patients diagnosed with anti-NMDA receptor encephalitis was 21 years (range 1–85) [11]. Approximately 40% were children [4, 6, 11]. The minimum age reported was 2 months [17].

## 3. Pathogenesis

The NMDA receptors require binding of glycine and glutamate simultaneously, as well as membrane depolarization for activation. The receptors are composed of NR1 and NR2 (A-D) subunits, which bind glycine and glutamate, respectively [18]. Excitotoxicity caused by the overactivity of NMDA receptors may lead to such disorders as stroke, epilepsy, Parkinson's disease, Alzheimer's disease, and Huntington's disease [19], while low activity of NMDA receptors may result in schizophrenia [20].

Anti-NMDAR antibodies bind selectively to synaptic and extrasynaptic NMDA receptors. Originally, the target of the antibodies was reported to be NR1/NR2B heteromer [1]. Subsequently, Dalmau et al. [4] demonstrated that the main epitope was in the N-terminal domain of the NR1 subunit. Then, a further study reported that amino acid 369 of the NR1 subunit was the main target region, and it did not change when the disorder relapsed [21].

The pathogenic role of anti-NMDAR antibodies has been established in both in vitro and in vivo models [4, 8, 22–24]. The specific binding between the antibodies and receptors leads to crosslinking and internalization of those receptors instead of apoptosis. Then, the number of NMDA receptors on the postsynaptic membrane decreases. The effect is titer-dependent and reversible after antibody titers decrease [4, 8]. In contrast, other glutamate receptors and synaptic proteins, number of synapses, presynaptic terminals, dendritic complexity, dendritic spines, and cell viability are unaffected. An experiment in female Lewis rats showed that the density of NMDA receptors in the hippocampus was dramatically reduced after they were infused with anti-NMDAR antibodies from patients. It was similar to the findings observed in the hippocampus of autopsied patients [8].

**Figure 1:**
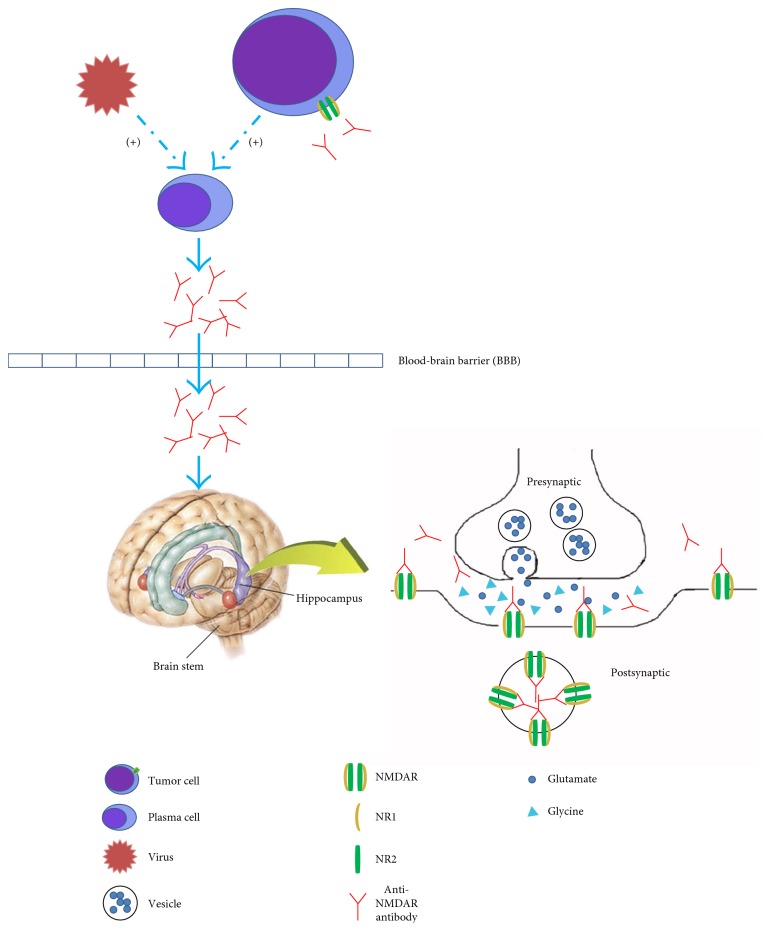
Possible pathogenesis of anti-NMDAR encephalitis. Anti-NMDAR antibodies synthesized by peripheral plasma cells pass through the broken blood-brain barrier (BBB). Tumors, which express NMDA receptors, as well as viral infections, may play a role in triggering the synthesis of anti-NMDAR antibodies. IL-6 and IL-17A might play an important role in the intrathecal antibody synthesis. NMDA receptors are expressed in many regions of the brain, including the hippocampus, brain stem, and neocortex. Anti-NMDAR antibodies bind selectively to synaptic and extrasynaptic NMDA receptors. The specific bind leads to crosslinking and internalization of those receptors. The number of NMDA receptors on the postsynaptic membrane decreases. The effect is titer-dependent and reversible after antibody titers decrease. Thus, anti-NMDAR antibodies lead to a specific, titer-dependent, and reversible reduction of NMDA receptors on postsynaptic dendrites which results in neuronal hypoactivity.

Thus, anti-NMDAR antibodies lead to a specific, titer-dependent, and reversible reduction of NMDA receptors on postsynaptic dendrites (Figure 1). Synaptic dysfunction caused by the loss of NMDA receptors results in the symptoms in patients with anti-NMDAR encephalitis, such as seizures, memory and learning deficits, and behavioral abnormities.

It has been reported that interleukin-6 (IL-6), interleukin-17A (IL-17A), and C-X-C motif chemokine 13 (CXCL13) were elevated in the CSF, while only interleukin-2 (IL-2) was increased in the serum of anti-NMDAR encephalitis patients [25]. Both IL-17A and IL-6 are proinflammatory cytokines. IL-17A could induce the expression of inflammatory gene in target cells [26], negatively regulate the tight junction molecules, and prompt leukocyte migrating across the blood-brain barrier (BBB) [27]. IL-6 could stimulate B-cell differentiation [28], enhance the survival of plasmablasts, and promote antibody production [29]. And IL-17A may trigger a positive-feedback loop for IL-6 signaling through signal transducer and activator of transcription 3 (STAT3) and nuclear factor (NF)-*κ*B [30]. In this way, the coactivation of IL-6 and IL-17A might play an important role in the intrathecal antibody synthesis of anti-NMDAR encephalitis. Further researches are needed to prove this finding. CXCL13 may be a potential biomarker of therapy response [31].

The increase of T cell-related cytokines (interferon-*γ* (INF-*γ*), tumor necrosis factor-*α* (TNF-*α*), and IL17A) in CSF has suggested that T-cell mechanisms may be also involved in the anti-NMDAR encephalitis [32], while humoral immune response has been proposed to be more relevant with this disease [33].

The triggers of the synthesis of anti-NMDAR antibodies include tumors, viral infections, and other unknown factors. Ovarian teratomas have been demonstrated to contain mature or immature neurons expressing NMDA receptors in both the autopsy and pathological studies, which reacted with patients' antibodies [33]. Furthermore, in the samples of teratomas from patients with anti-NMDAR encephalitis, inflammatory cell infiltrates were identified, including macrophages, T cells, B cells, and plasma cells, while were minimally present or absent in teratomas from patients without anti-NMDAR encephalitis [33, 34]. Thus, teratomas may play a role in triggering the synthesis of anti-NMDAR antibodies. However, it is unclear whether other tumors are triggers of anti-NMDAR encephalitis or unrelated coincidence. Anti-NMDAR encephalitis is often preceded by viral-like prodromal symptoms, and relapse occurs in 12% of patients with HSE although the clinical course of HSE is usually monophasic [35]. Anti-NMDAR encephalitis occurs also as post-HSE choreoathetosis [36]. There is a novel opinion that HSE plays a vital role in triggering the synthesis of anti-NMDAR antibodies, which has been confirmed by many investigators [3, 17, 37–40]. Those patients usually benefit from immunotherapy [38]. Thus, the intractable HSE or relapse post-HSE should catch the attention of clinicians, and detection of anti-NMDAR antibody should be performed no matter whether it was positive or not on the first episode.

## 4. Clinical Manifestations

The clinical manifestations are variable and sometimes easily misdiagnosed with viral encephalitis [39], psychosis [41–43], epilepsy, or other diseases, such as Hashimoto's encephalopathy [44] and Rasmussen syndrome [45]. Thus, it is a challenge to the psychiatrists, neurologists, and emergency physicians, as well as gynecologists and oncologists because of the association with teratoma or other tumors. Recognizing the characteristic features of anti-NMDAR encephalitis is vital to diagnose exactly and to permit a more timely treatment. The symptoms of the disorder are categorized in 8 groups: (1) psychiatric and behavioral symptoms, (2) seizures, (3) motor dysfunctions, (4) memory dysfunction, (5) speech disorders, (6) decrease in level of consciousness, (7) autonomic dysfunctions, and (8) central hypoventilation [11].

More than 80% of the patients with anti-NMDAR encephalitis have nonspecific symptoms with antecedent infection, such as fever, headache, or a viral-like manifestation (digestive-tract or upper respiratory-tract symptoms) [4, 16]. The percentage of patients who have antecedent infection in children is much lower [6, 46]. Most systemic symptoms cannot help us to distinguish anti-NMDAR encephalitis from other causes of encephalitis. Within a few days, usually less than two weeks, patients present with these 8 categories of symptoms, frequently psychiatric problems leading to the initial visit to psychiatrists [4].

1. Psychiatric and behavioral symptoms—approximately 80% develop obvious psychiatric and behavioral symptoms [4], including anxiety, irritability, insomnia, paranoia, aggression, auditory or visual hallucinations, sexual disinhibition, mania, cognitive disorder, and psychosis. In patients younger than 18 years old, those symptoms are less frequent. The difference may be attributable to the situation that the behavioral symptoms become difficult to detect in young children, because they often manifest with hyperactivity, irritability, or temper tantrums [6, 11]. In both sexes, psychiatric symptoms act as the most frequent initial symptom (54% in men, 67% in women) [16, 47]. Isolated psychiatric symptoms are rare (4%) but occur at the disease onset or during relapse. The isolated symptoms mainly include delusional thinking, mood disturbances (usually manic), and aggression [48].
2. Seizures—about 70% of anti-NMDAR cases present with seizures [2, 6]. In males, seizures are usually partial, while in females, generalized seizures are more common. Seizures are more frequent to act as initial symptom in adult male patients than in adult females [47, 49]. In children and adolescents, seizures are usually partial motor or complex seizures [6]. Even, anti-NMDAR encephalitis causes prolonged status epilepticus [50, 51], which carries a poor prognosis, with a mortality rate of 56% [52].
3. Motor dysfunctions—a wide range of abnormal movements are frequently observed, for example orofacial dyskinesias, chorea, ballismus, athetosis, rigidity, stereotyped movements, myorhythmia, or opisthotonus [2, 6, 53, 54]. Movement disorders are more common in children and atypical symptoms such as hemiparesis or cerebellar ataxia predominate in this age group [11]. Orofacial dyskinesias are the most frequent, including masticatory-like movements, grimacing, and forceful jaw opening and closing. Those symptoms result in lip and tongue injuries or broken teeth [4].
4. Memory dysfunction—short-term memory loss is common. However, it is usually underestimated, because language dysfunctions and psychiatric problems interfere with the evaluation of memory [4].
5. Speech disorders—language dysfunctions, including reduction of verbal output or mutism, echolalia (usually with echopraxia), mumbling, or perseveration, happen in more than 70% of patients with anti-NMDAR encephalitis [2, 5].
6. Decrease in level of consciousness: 88 of 100 patients presented with decreased consciousness during the first 3 weeks [4].
7. Autonomic dysfunctions—the most common manifestations of autonomic instability are hyperthermia, cardiac dysrhythmias (tachycardia or bradycardia) [55], hypersalivation, hypotension, hypertension, urinary incontinence, and sexual dysfunction [4]. Those dysfunctions occur frequently in the patients with anti-NMDAR encephalitis (69%) [4], especially in children (86%) [6], while they were not observed in viral encephalitis [2]. In children, tachycardia, hyperthermia, and hypertension occur predominantly [6].
8. Central hypoventilation—approximately 70% of patients develop hypoventilation [4]. About 20% of the patients require intubation because of central hypoventilation [6]. The symptom often happens when the patient becomes comatose but it also appears earlier when the level of consciousness is relatively preserved.

Psychiatric and behavioral problems are the most frequent initial symptoms [16, 47], especially in adults, while neurologic symptoms (especially seizures) occur initially as frequently as psychiatric symptoms in children [11, 36, 46]. Most cases (87%) develop four or more of those 8 classifications of symptoms four weeks after onset, while only 1% remain monosymptomatic [11], such as isolated psychosis [48], abnormal movements [56, 57], or seizures [58]. At the peak of anti-NMDAR encephalitis, each of the following symptoms occur in more than 50% of the cases: psychiatric and behavioral problems, seizures, movement disorders, cognitive dysfunctions (anterograde amnesia, alteration of mental status, and speech disorder), and decreased level of consciousness [47]. Symptom presentation is different between adults and children (“more psychiatric in adults”, “more neurological in children”) [11]. Memory loss, as well as central hypoventilation, is observed more frequently in adults, while motor dysfunctions and ataxia predominate in children. Within the first month, most cases progress to a similar spectrum of manifestations regardless of age [11, 36, 59].

In addition to those characteristic manifestations, central neurogenic hyperventilation has been reported in such patients [60]. The cranial nerves are also involved in anti-NMDAR encephalitis. Moreover, cervical rigidity is less frequent to appear [2]. Some patients suffer from sleep dysfunction, such as hypersomnia and inversion of sleep patterns [4].

The modified Rankin scale (mRS) has been used to assess the neurological status of patients with anti-NMDAR encephalitis [61]. In a large sample cohort study, the disease severity showed that 87% of patients had a maximum mRS of 5, and 77% needed the support of ICU. On the other hand, spontaneous improvement also occurred in several patients [11]. The clinical features of anti-NMDAR encephalitis in adults and children are presented in Table 1.

## 5. Association with Tumors

Anti-NMDAR encephalitis has been found to associate with ovarian teratoma in young women [15]. The frequency of an underlying tumor is dependent on sex, age, and ethnic background of the patients [4, 6, 11]. An underlying neoplasm could be found in a large group of the patients (38%), especially in women (46%). It is rarely in girls younger than 12 years (6%) and male patients (6%). The presence of a tumor predominates in cases between 12 and 45 years [11]. Tumor is usually less discovered in the younger patients [5, 6]. Twenty-three percent of the patients older than 45 years have underlying tumors, which are usually carcinomas rather than teratomas [62]. Black patients are more likely to have an underlying tumor than other ethnic groups [5]. Ovarian teratoma, most of which is mature [4], is the most common underlying tumor (94%). Extraovarian teratoma (2%) and other tumors (4%, such as tumors of the lung, breast, testis, ovary, uterus, thymus, and pancreas) are also detected. Those tumors other than teratomas are often detected in patients older than 45 years [11, 63–65].

Hepatic lesions, which were focal nodular hyperplasia by biopsy, were reported in a 12-year-old girl with anti-NMDAR encephalitis. The association between anti-NMDAR encephalitis and liver tumors is unclear [66]. It is recently reported that anti-NMDAR encephalitis developed shortly after receiving combination treatment with immune checkpoint inhibitors (nivolumab and ipilimumab) for metastatic melanoma [67]. Antibody-negative limbic encephalitis occurred one year after starting pembrolizumab for malignant melanoma [68]. Immune checkpoint inhibition may contribute to the development of immune responses against neuronal antigens, causing autoimmune encephalitis. Another possibility is that it belongs to the classic paraneoplastic neurologic disorders (PNDs) associated with metastatic melanoma [69]. Further researches are required to confirm if there is causality between melanomas and autoimmune encephalitis or immune checkpoint inhibition can trigger autoimmune encephalitis.

On the other hand, some patients with teratoma developed several kinds of encephalitis without NMDAR antibodies. Among those forms of encephalitis, a syndrome with brainstem-cerebellar symptoms stood out. In those patients without NMDAR antibodies, psychosis and behavioral change were less likely to act as the initial symptom, and other symptoms except psychosis and behavioral change (such as dyskinesias) were uncommon [70].

## 6. Laboratory Findings and Imaging Manifestations

In order to make a precise diagnosis, especially in the initial phase of anti-NMDAR encephalitis, the rational assistant examinations are necessary. The conventional CSF test, the examinations by magnetic resonance imaging (MRI), and electroencephalogram (EEG) could provide the valuable information on anti-NMDAR encephalitis.

1. CSF test—abnormal alterations in CSF are seen in more than 90% of patients. These abnormities include mild-to-moderate lymphocytic pleocytosis (90%), mild increase of protein concentration (30%), and CSF-specific oligoclonal bands (60%) [4]. The median value of white blood cells (23/mm^3^) is significantly lower than that in cases of viral etiologies. The protein level with a median of 24 mg/dl is also significantly lower. The glucose value is usually within normal range [2]. The oligoclonal bands are detected even when routine CSF examinations are normal. In the early stage, few oligoclonal bands are observed but become more prominent later in the disease course [71]. The changed profile of CSF in children resembles that in adults [6]. The incidences of CSF abnormities (pleocytosis or increase of protein concentration) reported in China [16, 72, 73] were lower than that reported by Dalmau et al. [4]. The distinction may be attributed to the difference in sample size, population composition, or ethnic background which needs further studies to prove.
2. MRI manifestations—the results of routine MRI examinations in the brain are abnormal only in 30%–50% of patients with anti-NMDAR encephalitis [2, 4, 11]. Increased signals on fluid-attenuated inversion recovery (FLAIR) and/or on T2 sequence are observed frequently in the cortical and subcortical regions and hippocampus, sometimes in the basal ganglia, posterior fossa, or medial temporal regions. The cortical-meningeal enhancement with gadolinium is less frequent and transient. Most of the abnormalities in MRI manifestations are often mild, transient, and nonspecific [2]. Multifocal or extensive demyelinating changes are also found, which suggests that anti-NMDAR encephalitis patients may develop episodes of demyelinating disorders simultaneously or separately [74]. Despite normal manifestations in routine MRI, extensive alterations of white matter integrity and substantial changes of functional connectivity are visible in patients with anti-NMDAR encephalitis using diffusion tensor imaging and functional MRI. The changes of white matter are most frequently observed in the cingulum and these changes are correlated with disease severity [75]. Normal MRI findings may change after a sudden hypoxic period caused by seizures, respiratory failure or cardiac arrest, because some regions become hypermetabolic and susceptible to hypoxia [76].
3. EEG—EEG is abnormal in 90% or even more patients with anti-NMDAR encephalitis. Most patients develop extensive EEG abnormalities characterized by focal or generalized slow activity with or without epileptic discharges [2, 4, 6, 11]. Extreme delta brush is regarded as a unique electrographic pattern of anti-NMDAR encephalitis. It is characterized by generalized rhythmic delta activity at 1–3 Hz with superimposed rhythmic 20–30 Hz beta frequency activity. The pattern was previously described in 30% of 23 adult patients undergoing continuous EEG monitoring. The delta brush is related to a more prolonged course and should raise consideration of anti-NMDAR encephalitis [77]. The EEG abnormities are often subclinical, while some movement disorders suggestive of seizures have no EEG correlation [6]. EEG will be helpful to distinguish between seizures and movement disorders.

## 7. Anti-NMDAR Antibodies

Antibodies of the IgG class against subunit NR1 of NMDAR were first demonstrated in connection with anti-NMDAR encephalitis as the indicator of this disorder [4]. The pathogenic role of these antibodies has been demonstrated in cultured neurons and in vivo models [7, 23]. The technologic methods of detection of anti-NMDAR antibodies comprise immunohistochemistry and cell-based assay (CBA) with fixed or live cells, which are reliable antibody-testing methods. NMDAR antibodies could be detected using the techniques in CSF (both sensitivity and specificity as 100%). However, it is less sensitive and specific using CBA to detect the antibodies in serum, in which the misdiagnosis rate is 13%. Even if both of those techniques are used, the missed diagnosis rate remains 7% [21]. To avoid misdiagnosis as other diseases, such as Creutzfeldt-Jakob disease [78] and schizophrenia [79], it is recommended that either using CBA detects the NMDAR antibodies in both serum and CSF or applying both CBA and immunohistochemistry techniques detect the antibodies in serum.

Seropositive findings are more likely to be observed in patients with teratoma than those without a tumor. In addition, there is an association between high levels of antibody and the teratoma and/or poor outcome. Over time, the antibody titer may decrease regardless of outcome [4, 21]. The patients with an early decrease of antibody levels in CSF within the first months tend to have a good outcome [21]. After clinical recovery, CSF and serum from some patients may remain antibody positive [21, 80, 81]. The level change of the antibodies in CSF is more closely related with clinical relapses than that in serum [21].

Regardless of immunoglobulin class (IgM, IgA, and IgG), all circulating autoantibodies against the NR1 subunit of NMDA receptors may have pathogenic potential on access to the brain in the condition of increased BBB permeability [82]. In contrast to IgG antibody which has high disease specificity of anti-NMDAR encephalitis [21], IgA and IgM antibodies may be elevated in healthy individuals and many disease carriers, ranging from major depression and schizophrenia [42] to hypertension, diabetes and stroke [83] and to multiple sclerosis (MS) [84], dementia [85, 86], Alzheimer's and Parkinson's disease [87, 88]. Those patients with high levels of IgA and IgM antibodies may potentially benefit from immunotherapy.

## 8. Diagnosis

Recently, the diagnostic criteria for anti-NMDAR encephalitis have been made by Graus et al. [89], which are based on the clinical manifestations, evidences of CSF, brain MRI and EEG, and the antibodies against the NR1 subunit of NMDARs in the CSF and/or serum.

The diagnosis of probable anti-NMDAR encephalitis can be made when all three of the following conditions have been reached: (1) at least four of the six major groups of symptoms occur within 3 months, including behavioral (psychiatric) abnormity or cognitive dysfunction, speech dysfunction (pressured speech, reduction of verbal output, and mutism), seizures, motor dysfunction, decreased level of consciousness, autonomic instability or central hypoventilation—cases with three of the above groups of symptoms together with a systemic teratoma can also be diagnosed; (2) at least one of the following laboratory findings: EEG abnormity (focal or diffuse slow or disorganized activity, extreme delta brush, or epileptic activity) and CSF abnormity (pleocytosis or oligoclonal bands); (3) exclude other disorders.

The diagnosis can be definite when one or more of the six groups of symptoms are present and IgG antibodies against the GluN1 subunit of the NMDA receptor are detected. Also, reasonable exclusion of other disorders is necessary. Antibody testing should include CSF analysis. If only serum is available, in addition to CBA, live neurons or tissue immunohistochemistry should be used as confirmatory test.

## 9. Anti-NMDAR Encephalitis Coexistences with Demyelinating Disorders

Anti-NMDAR encephalitis coexisting with demyelinating diseases may occur in some individuals sequentially or simultaneously. Acute demyelinating encephalomyelitis (ADEM), neuromyelitis optica (NMO), optic neuritis, myelitis, MS, prominent brainstem dysfunction, or other demyelinating disorders with the anti-NMDAR encephalitis have been reported [74, 90–95].

In a study of 691 patients suffering from anti-NMDAR encephalitis with the median age as 27 years (range 4–62 years), 23 patients (3.3%) manifested with obvious clinical and/or MRI features suggesting demyelination [74]. In 12 of those 23 patients, anti-NMDAR encephalitis was identified before or after the independent episodes of demyelinating disorders evidenced by detections of antibodies against aquaporin-4 (AQP4) and myelin oligodendrocyte glycoprotein (MOG) using CBA and immunohistochemistry, including neuromyelitis optica spectrum disorder (NMOSD) and brainstem or multifocal demyelinating syndromes. In 5 cases with NMOSD, 4 were anti-AQP4 antibody positive. All 7 cases with brainstem or multifocal demyelinating syndromes were anti-MOG antibody positive. The other eleven patients developed anti-NMDAR encephalitis and demyelinating features simultaneously (5 anti-AQP4 antibody positive, 2 anti-MOG antibody positive). Teratoma was less frequent in those 23 patients with overlapping syndromes than anti-NMDAR antibody only controls. Clinical symptoms in majority of patients with anti-NMDAR encephalitis coexisting with demyelinating diseases have improved after immunotherapy. More intensive care was needed in patients with demyelinating episodes and those patients remained more residual deficits [74].

A previous healthy 44-year-old Chinese woman who was followed up for 8 years in the First Hospital of Jilin University developed anti-NMDAR encephalitis coexisting with NMOSD with negative anti-AQP4 antibody in serum and CSF (submission).

Anti-NMDAR encephalitis in coexistence with demyelinating diseases made the clinical manifestations too complex to recognize. Therefore, the rational assistant examinations, especially detection of anti-NMDAR antibodies and other antibodies related to diagnosis of demyelinatory disorders in CSF and/or serum are conducive to early diagnosis.

In practice, clinicians should be aware that overlapping syndromes may occur and specific antibody testing should be performed when patients with anti-NMDAR encephalitis develop demyelinating features, and patients with NMOSD or other demyelinating disorders develop atypical symptoms (e.g., seizures, psychosis).

The exact contribution of these antibodies (NMDAR, AQP4, MOG, or unknown antibodies) to myelin dysfunction is unclear, but it should be noted that there are NMDA receptors on oligodendrocytes [96] that are the target cells in most demyelinatory disorders in the CNS. Thus, future studies are needed to determine whether NMDA receptors on oligodendrocytes could be affected by those antibodies.

A recent study reported a 22-year-old male with human immunodeficiency virus (HIV) infection manifested with obvious psychiatric symptoms and had anti-NMDAR antibodies in his serum using CBA and immunohistochemistry [97]. This patient might develop an overlapping syndrome. Another possibility is that infection of HIV could trigger anti-NMDAR encephalitis. To date, the similar reports are rare and underlying mechanism is still unknown.

## 10. Treatment, Relapses, and Outcome

**Figure 2:**
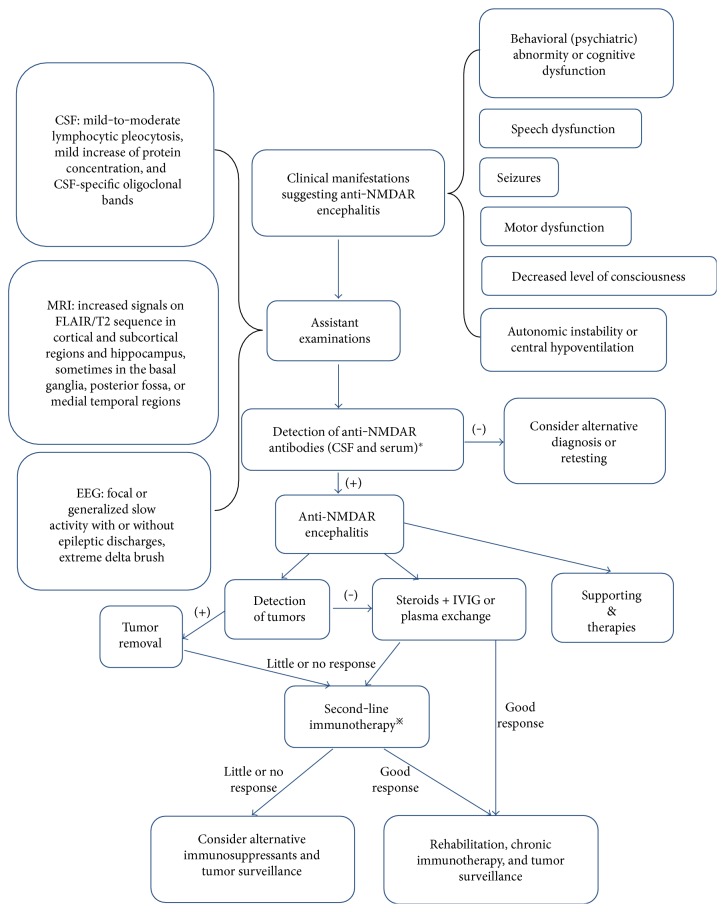
Procedure of diagnosis and treatment of anti-NMDAR encephalitis. ∗ indicates that detection of antibodies should include CSF analysis. If only serum is available, in addition to cell-based assay (CBA), live neurons or tissue immunohistochemistry should be used as confirmatory test. ^&^Supporting therapies include antiepileptic and antipsychotic treatments, respiratory and cardiac support, management of blood pressure and temperature, and prevention of deep venous thrombosis (DVT) and bedsore. ^※^Cyclophosphamide, rituximab, or both.

Despite the severity of anti-NMDAR encephalitis, patients often get improvement with the support of multidisciplinary care, including immunotherapy, surgery, ICU support, and sometimes prolonged hospitalizations [4]. Immunotherapy and detection and removal of a teratoma should be initially focused on (Figure 2 presents the procedure of diagnosis and treatment of anti-NMDAR encephalitis). First-line immunotherapy consists of steroids, intravenous immunoglobulin (IVIG), and/or plasma exchange (PE), which could be used alone or combined [11]. If tumors are found, the surgical resection should be taken into account [1, 98]. When diagnosis is delayed, or patients do not have a tumor, or the first-line immunotherapy fails, additional treatment with second-line immunotherapy is usually applied [5, 11], which includes cyclophosphamide, rituximab, azathioprine, mycophenolate mofetil, methotrexate, and so on [99, 100]. Supporting therapies play an important role, for example antiepileptic and antipsychotic treatment, respiratory and cardiac support, management of blood pressure and temperature, and prevention of deep venous thrombosis (DVT) and bedsore [46, 101]. After the acute phase, many patients need rehabilitation therapies, such as occupational and physical therapy, as well as therapies for dysphagia and speech [46, 102].

A drastically different outcome occurred in identical twin sisters with anti-NMDAR encephalitis. Neither of them responded to immunotherapy. Imaging examinations showed normal-appearing ovaries, which were confirmed by autopsy or pathology. The first twin received immunotherapy only (prednisone, cyclophosphamide, rituximab, and plasmapheresis) and died from the disease, while the second twin accepted a bilateral salpingo-oophorectomy and recovered gradually, except for slight memory deficits. That unique clinical scenario suggests that patients who fail to respond to first- and second-line immunotherapy may benefit from the removal of normal-appearing ovaries [103]. Additionally, when patients with anti-NMDAR encephalitis do not improve after first- and second-line treatments, local intrathecal treatment with methotrexate and/or methylprednisolone may be a promising alternative therapy [104, 105]. Furthermore, coenzyme Q10 may have a beneficial role in treatment of anti-NMDAR encephalitis [106].

In a multi-institutional study including 577 patients (1–85 years, median 21 years) [11], 501 patients were followed up to assess the therapeutic effects and outcome. 472 (94%) cases were treated with first-line immunotherapy or tumor removal, approximately 50% of whom improved within four weeks. In the remaining 221 patients who failed to respond to first-line treatment, 125 (57%) patients received second-line immunotherapy (rituximab and cyclophosphamide) and got better outcome than those who did not. Of those 501 patients, 79% got good outcome (mRS 0–2) within the first 24 months (median 6 months). 81% had a good outcome at a 24-month follow-up, and some patients continued to improve thereafter. In multivariable analysis, predictors of good outcome included early treatment and no need for ICU. The use of second-line immunotherapy was identified as an additional factor for good outcome by multivariable analysis. Longer follow-up was associated with better outcome.

In another study including 105 patients with anti-NMDAR encephalitis, patients with a tumor (mostly teratoma) were more likely to achieve substantial improvement after first-line immunotherapy and tumor resection than those without a tumor (80% versus 48%). Second-line immunotherapy was needed more often in those patients without a tumor. The final outcome was quite similar in patients with or without a tumor [5].

The findings have demonstrated that early and aggressive immunotherapy and tumor resection (if present) contribute to achieve favorable outcomes, which is in line with other reports [14, 71, 107].

Dalmau et al. has reported that recovery of anti-NMDAR encephalitis often develops as a multistage process that occurs in the reverse order of symptom appearance [5]. The median hospital admission is 2.5 months [4], but longer hospitalizations in rehabilitation centers may be needed for many patients [46, 102, 108]. Approximately 50% of patients achieve full recovery, while 28% and 18% remain mild and severe deficits, respectively [4]. The mortality rate of this disorder is about 6% [4, 11]. There is an association between high antibody-titers and poor outcome. Patients with an early decrease of antibody titers in CSF within the first four weeks of the disease tend to have a good outcome [21].

Relapse is defined as worsening of symptoms or the new onset occurring after more than 2 months of stabilization or improvement. Some patients has one or multiple relapses, which represents a 12% risk of relapses within 2 years [11]. Relapses predominantly affect those patients without tumors or who are treated with delayed immunotherapy and tumor resection (if present) [4, 10, 11]. The first relapse may occur many years after the initial episode (range 0.5–13 years, median 2 years). More than 50% of the relapses may present with partial aspects of the previous episodes, and they do not add residual deficit [10, 11].

Responses to immune treatment in children and teenagers (younger than 18 years) are slow and variable [6]. It is reported that 75%–85% have full or substantial recovery after immunotherapy or tumor resection [6, 36, 46], which is similar to that of adults [11]. Relapses occur in 15%–25% of children with anti-NMDAR encephalitis [6, 46].

In patients older than 45 years, the outcome is usually less favorable than in younger patients even though the clinical manifestations are less severe. These may account for the discrepancy: (1) in this age group (older than 45 years), underlying tumors are less common, but if present, they are usually carcinomas rather than teratomas; (2) delays in diagnosis and treatment are more frequent. Except for no need for ICU, early treatment, and longer follow-up, younger age is also a predictor of good outcome [62].

## 11. Conclusion

Anti-NMDAR encephalitis mainly affects young women with ovarian teratomas but also occurs in other subjects. It is a potentially lethal but treatable autoimmune disorder characterized by obvious psychiatric and neurologic manifestations. Laboratory and imaging as well as EEG examinations often show abnormalities. The diagnosis is based on the detection of IgG antibodies against the GluN1 subunit of NMDA receptors in CSF and/or serum. Anti-NMDAR encephalitis in coexistence with demyelinating disorders makes the disease more difficult to recognize.

Prompt immunotherapy, complete tumor resection, and ICU support contribute to a favorable outcome. Future studies should focus on exploring the associations with tumors and infectious triggers, underlying mechanisms of anti-NMDAR encephalitis and overlapping syndrome, and developing new therapies.

## Figures and Tables

**Table 1:** Clinical features of anti-NMDAR encephalitis in adults and children.

	Adults	Children
Antecedent infection (0–2 weeks)	More than 80% of patients; fever, headache, digestive-tract or upper respiratory-tract symptoms	Less common
Psychiatric and behavioral symptoms	About 80% of the cases; anxiety, irritability, insomnia, paranoia, aggression, auditory or visual hallucinations, sexual disinhibition, mania, cognitive disorder and psychosis; isolated psychiatric symptoms are rare	Less common
Seizures	About 70% of the cases; usually partial in males, and generalized in females; prolonged status epilepticus may occur	Usually partial motor or complex seizures; initially as frequently as psychiatric symptoms
Motor dysfunctions	Orofacial dyskinesias, chorea, ballismus, athetosis, rigidity, stereotyped movements, myorhythmia, or opisthotonus	More common; atypical symptoms such as hemiparesis or cerebellar ataxia predominate in this age group
Memory dysfunction	Short-term memory loss	Less common
Speech disorders	More than 70% of patients; reduction of verbal output or mutism, echolalia (usually with echopraxia), mumbling, or perseveration	
Decrease in level of consciousness	88% of patients during the first 3 weeks	
Autonomic dysfunctions	About 70% of the cases; hyperthermia, cardiac dysrhythmias (tachycardia or bradycardia), hypersalivation, hypotension, hypertension, urinary incontinence, and sexual dysfunction	More common; predominantly tachycardia, hyperthermia, and hypertension
Central hypoventilation	Approximately 70% of patients	Less common

## Abbreviations

ADEM: Acute demyelinating encephalomyelitis
AQP4: Aquaporin-4
BBB: Blood-brain barrier
CBA: Cell-based assay
CSF: Cerebrospinal fluid
CXCL13: C-X-C motif chemokine 13
DVT: Deep venous thrombosis
EEG: Electroencephalogram
FLAIR: Fluid-attenuated inversion recovery
HIV: Human immunodeficiency virus
HSE: Herpes simplex encephalitis
HSV-1: Herpes simplex type 1
ICU: Intensive care unit
IL-17A: Interleukin-17A
IL-2: Interleukin-2
IL-6: Interleukin-6
INF-*γ*: Interferon-*γ*
IVIG: Intravenous immunoglobulin
MOG: Myelin oligodendrocyte glycoprotein
MRI: Magnetic resonance imaging
mRS: Modified Rankin scale
MS: Multiple sclerosis
NF: Nuclear factor
NMDAR: N-Methyl-D-aspartate receptor
NMO: Neuromyelitis optica
NMOSD: Neuromyelitis optica spectrum disorder
PE: Plasma exchange
PNDs: Paraneoplastic neurologic disorders
STAT3: Signal transducer and activator of transcription 3
TNF-*α*: Tumor necrosis factor-*α*
VZV: Varicella-zoster virus
WNV: West Nile virus.

## Additional Points

*Availability of Data and Materials*. The data supporting the conclusions of this review are included within the “References” section.
